# Combined Hamartoma of the Retina and Retinal Pigment Epithelium: A Literature Review and Case Series

**DOI:** 10.7759/cureus.96688

**Published:** 2025-11-12

**Authors:** Inês Ludovico, Patrícia Silva, Lívio Costa, Rita Flores, Rita Anjos

**Affiliations:** 1 Ophthalmology, Unidade Local de Saúde de São José, Lisbon, PRT

**Keywords:** epirretinal membrane, fluorescein angiography, fundus autofluorescence, hamartoma of retina and retinal pigment epithelium, neurofibromatosis, optical coherence tomography (oct)

## Abstract

We present a case series of two young patients referred to our Ophthalmology Department with a progressive decrease in visual acuity of their left eye, where multimodal imaging findings led us to the diagnosis of combined hamartoma of the retina and retinal pigment epithelium (CHR-RPE). Based on a literature review, we discuss the different multimodal imaging strategies, differential diagnosis, and management of CHR-RPE, a rare benign congenital lesion that can mimic malignant intraocular tumors. CHR-RPE has a challenging differential diagnosis with entities such as choroidal melanoma, nevus, retinoblastoma, and astrocytoma. A correct diagnosis approach is essential to avoid unnecessary invasive procedures. Multimodal imaging plays a crucial role: Optical coherence tomography (OCT) reveals epiretinal membranes (ERM) and retinal disorganization, and fluorescein angiography (FA) demonstrates vascular tortuosity and leakage in advanced disease. Management is usually conservative with strict follow-up. Surgical intervention, including vitrectomy and membrane peeling, remains controversial due to the risk of damaging dysplastic retina. Reported outcomes are variable, although selected cases showed visual improvement, particularly when combined with adjunctive enzymatic therapy.

## Introduction

Combined hamartoma of the retina and retinal pigment epithelium (CHR-RPE) is a rare congenital mass composed of disorganized glial, vascular, and melanocytic tissue affecting the neurosensory retina, retinal vessels, and retinal pigment epithelium (RPE) [1]. These lesions most commonly occur in a peripapillary location, although macular involvement has also been reported. Concurrent vitreoretinal traction may lead to retinal folds, foveal ectopia, foveoschisis, tractional retinal detachment, and, ultimately, vision loss.

Caucasian males appear to be at higher risk [2]. Gass was the first to describe the CHR-RPE in 1973, as a slightly elevated, charcoal grey mass involving the RPE, retina, and overlying vitreous, blending imperceptibly with surrounding RPE and extending in a fanlike projection toward the periphery [3].

Although the exact origin of these lesions remains uncertain, a recent study by Pujari et al. suggested that they may arise from undifferentiated ectopic progenitor cells destined to become the RPE but failing to complete differentiation, resulting in hyperplasia and accumulation within the neurosensory retina [4]. Most cases of CHR-RPE are unilateral and occur as isolated findings; however, some have been associated with systemic diseases, most notably Neurofibromatosis type 1 and 2, particularly when lesions are bilateral [2].

The main clinical challenge associated with CHR-RPE lies in establishing the differential diagnosis from other retinal lesions, such as melanoma and nevi of the choroid, retinoblastoma, retinal hemangioma, and astrocytoma [2].

Accurate distinction is essential to avoid unnecessary invasive procedures. In this context, imaging examinations, particularly OCT, autofluorescence (AF), and FA, are crucial for characterizing the lesion’s morphology and guiding the therapeutic approach.

## Case presentation

Case 1

**Figure 1:**
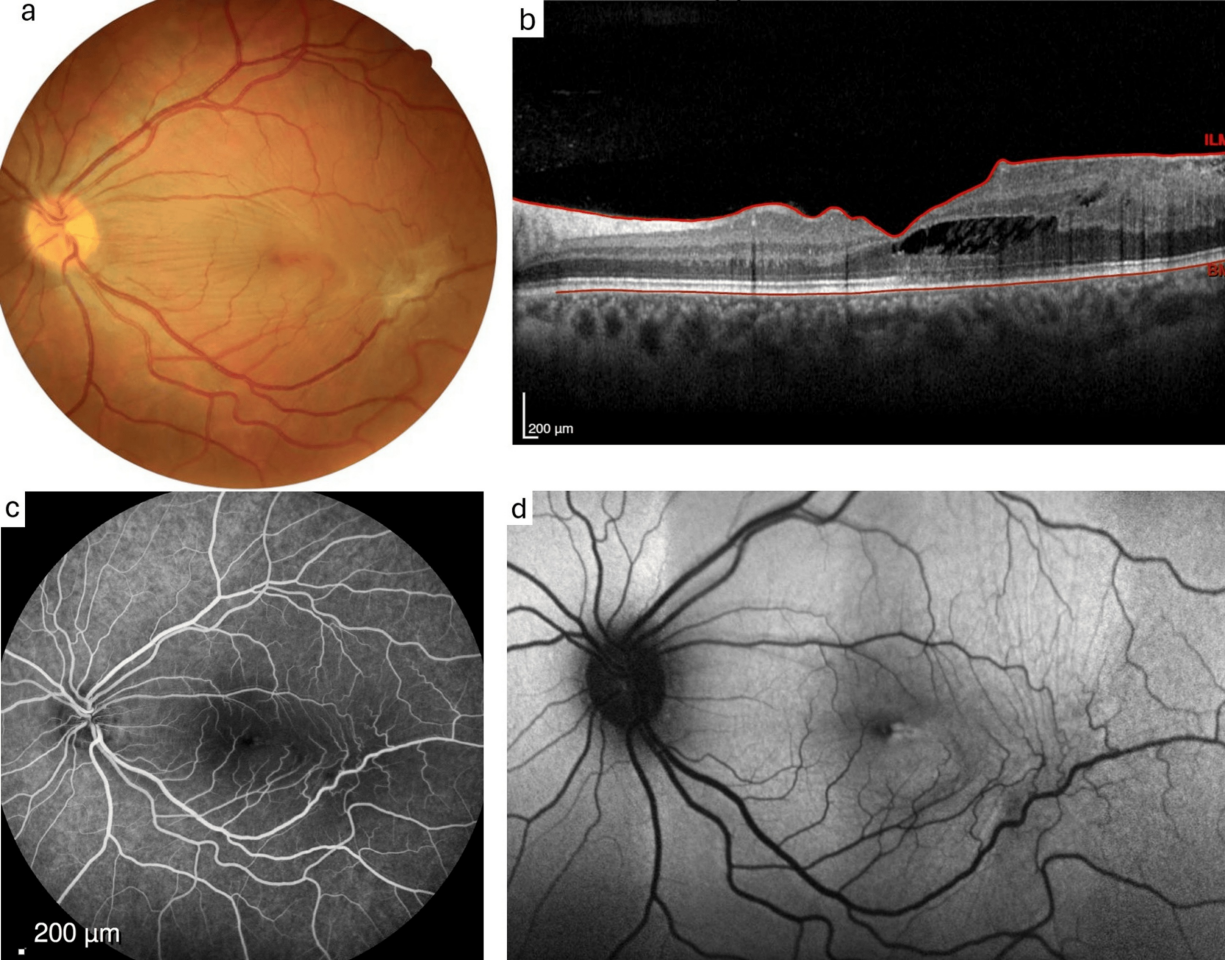
Multimodal imaging of the left eye a: Color fundus photograph showing a slightly elevated lesion measuring approximately 11 x 11 mm in the inferotemporal region. b: OCT showing the presence of an ERM with macular traction leading to foveoschisis. c: Fluorescein angiography showing no signs of vascular leakage. d: Fundus autofluorescence demonstrates hypoautofluorescence in inferotemporal region.

A 26-year-old Caucasian male was referred to our center with a progressive decrease in visual acuity of the left eye. Ophthalmological examination revealed a best-corrected visual acuity (BCVA) of 10/10 in the right eye (RE) and 7/10 in the left eye (LE). Biomicroscopy and intraocular pressure were normal bilaterally. While fundus examination of the RE was unremarkable, a slightly elevated lesion measuring approximately 11 × 11 mm was identified in the inferotemporal region of the LE, with a bluish-gray coloration and almost complete fibroglial tissue coverage (Figure 1a). OCT demonstrated the presence of an epiretinal membrane (ERM) with macular traction leading to foveoschisis, as well as inner retinal hyperreflectivity with posterior shadowing, significant cytoarchitectural distortion, and central macular thickness of 353 μm (Figure 1b). FA showed no evidence of vascular leakage (Figure 1c), while AF revealed a hypoautofluorescent area in the inferotemporal region of the macula (Figure 1d).

Case 2

**Figure 2:**
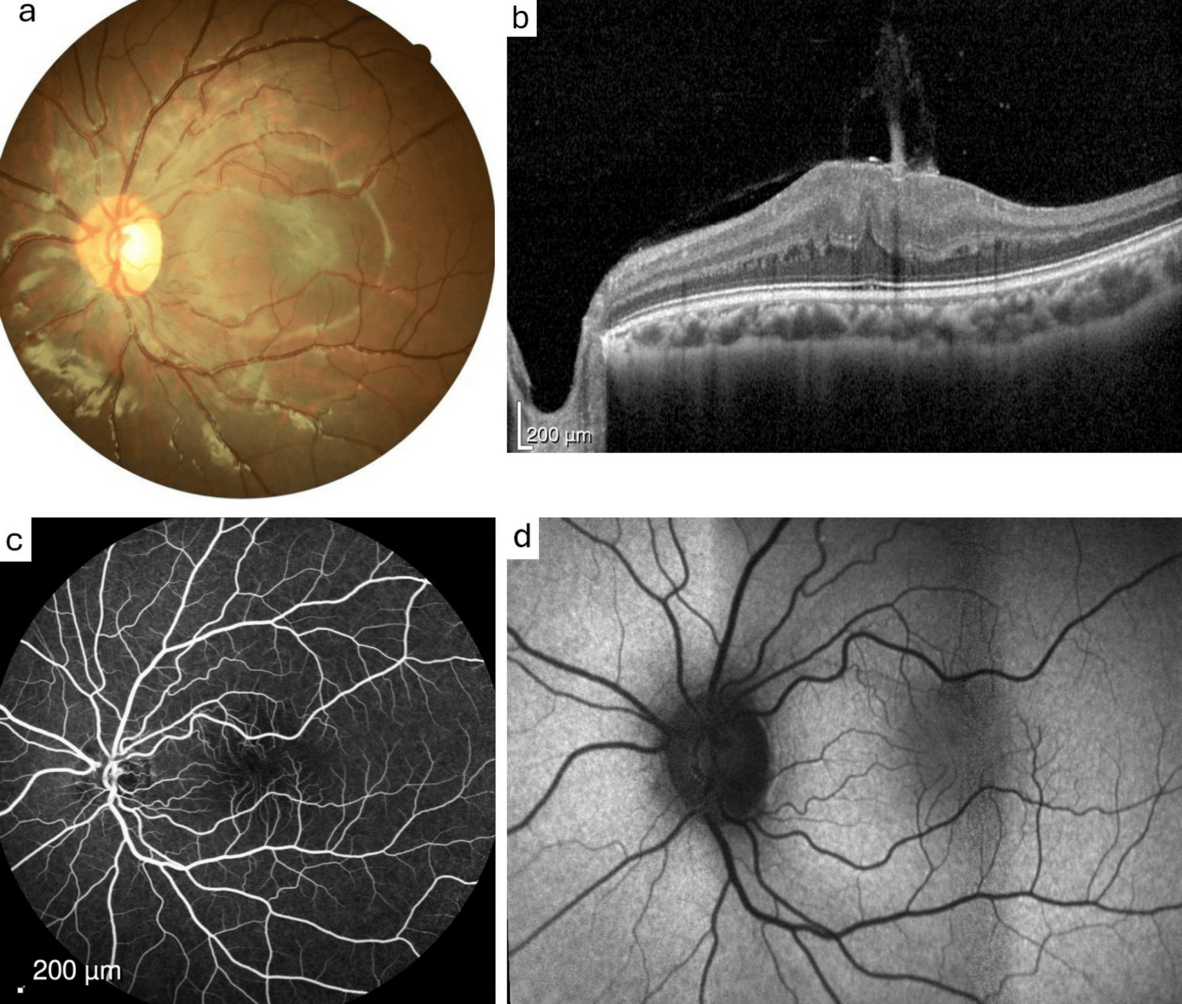
Multimodal imaging of the patient's left eye a: Color fundus photograph showing a thickened, elevated lesion temporal to the macula with apparent posterior hyaloid thickening. b: OCT demonstrated inner retinal thickening and hyperreflectivity. c: Fluorescein angiography showing no filling delay or vascular tortuosity. d: Fundus autofluorescence showing mixed hypo- and hyperautofluorescent pattern in the temporal macular region.

A 14-year-old female with no relevant family history was referred to our center due to decreased visual acuity in the left eye. Ophthalmological examination revealed a BCVA of 10/10 in the RE and 3/10 in the LE. Biomicroscopy and intraocular pressure were normal bilaterally. Fundus examination of the LE revealed a thickened, elevated lesion temporal to the macula with apparent posterior hyaloid thickening (Figure 2a). OCT demonstrated inner retinal thickening and hyperreflectivity, with a central macular thickness of 514 μm in the LE (Figure 2b). FA showed no filling delay, vascular tortuosity, or alterations (Figure 2c), and AF revealed a mixed hypo- and hyperautofluorescent pattern in the temporal region of the macula (Figure 2d).

## Discussion

CHR-RPE remains a challenging diagnosis due to its variable presentation and resemblance to malignant lesions. In our cases, multimodal imaging was essential for accurate characterization, consistent with findings reported in previous studies. The mean age of presentation differs between the studies, from a median age of seven months for macular and eight months for extramacular tumors in Shields et al.'s study to 13 years in Yassin et al.'s study (RA1) [5]. Although CHR-RPE is most frequently an isolated unilateral finding, associations with systemic disorders, most notably neurofibromatosis type 1 and 2 and, less frequently, tuberous sclerosis, have been described. In addition, choroidal neovascularization, secondary ERM formation, macular hole, optic nerve head pits, and optic disc drusen have been reported as concurrent findings [6].

Color fundus photography is valuable for documenting lesion morphology and progression, particularly regarding pigmentation and basal dimensions [7]. OCT plays a key role in both the detection and follow-up of ERM that often accompany this rare entity. Typical findings include inner retinal thickening and hyperreflectivity, disorganization of the retinal layers, cystoid macular changes, loss of the RPE and ellipsoid zone, and variable posterior shadowing [8,9]. FA often reveals vascular tortuosity, though staining, delayed leakage, ischemia, and neovascularization may occur in complicated cases [7]. Ocular ultrasonography can be a valuable tool in excluding malignant entities. The absence of a dome-shaped elevation and choroidal excavation helps to rule out choroidal melanoma, while the absence of calcifications may help to exclude retinoblastoma [7].

Given its generally stable behavior, CHR-RPE is usually managed conservatively with strict multimodal imaging follow-up to detect potential complications such as ERM formation, retinal holes, retinoschisis, choroidal neovascularization (CNV), retinal neovascularization, hemovitreous, and retinal detachment.

Surgical management, consisting of pars plana vitrectomy with ERM peeling, remains controversial. Experts note that the overlying glial membrane is often interwoven with the dysplastic retina, making complete removal technically challenging and increasing the risk of retinal damage. A comprehensive review by Zhang et al. summarized 13 small case reports including 43 patients, showing that surgical intervention generally resulted in a favorable visual outcome, with a success rate exceeding 70%. In 2009, Zhang performed careful peeling of the ERM while preserving the integrity of the hamartoma in five patients. However, the results were not promising because the BCVA improved only in three out of five (60%) patients, suggesting variable benefits. Zhang et al. hypothesized that another reason for the negative results could be the lack of timely amblyopia therapy in some pediatric patients [10].

Cohn et al. proposed the use of autologous plasmin enzyme as a potential surgical adjuvant to facilitate ERM removal. The enzyme promotes cleavage at the vitreoretinal interface, thereby easing membrane dissection and reducing tractional stress on the retina. Although preliminary outcomes were encouraging, larger cohort studies are still required to validate the efficacy and safety of autologous plasmin enzyme in CHR-RPE-associated ERM surgery [11].

## Conclusions

CHR-RPE is a rare benign congenital lesion whose clinical presentation can range from an incidental finding to a lesion mimicking intraocular malignancy. Accurate recognition is essential to avoid diagnostic errors with potentially serious consequences, particularly in pediatric patients. Advances in imaging techniques, such as OCT, have greatly enhanced morphological characterization, thereby improving diagnostic precision and guiding appropriate management of this uncommon entity.
